# Grapevine (*Vitis* spp.) rootstock stilbenoid associations with host resistance to and induction by root knot nematodes, *Meloidogyne incognita*

**DOI:** 10.1186/s13104-020-05201-3

**Published:** 2020-07-29

**Authors:** Christopher M. Wallis

**Affiliations:** USDA-ARS San Joaquin Valley Agricultural Sciences Center, Crop Diseases, Pests and Genetics Research Unit, 9611 S. Riverbend Ave, Parlier, CA 93648 USA

**Keywords:** Induced defense responses, Phenolics, Plant host resistance, Stilbenoids, *Vitis champinii*, *Vitis rotundifolia*, *Vitis riparia*, *Vitis vinifera*

## Abstract

**Objective:**

The root knot nematodes (RKN) *Meloidogyne incognita* can severely reduce grapevine yields over time. Grapevine rootstocks have been developed from wild *Vitis* species that provide resistance to nematode infections. However, the potential biochemical or mechanical mechanisms of resistance have not been thoroughly explored. Therefore, this study measured levels of stilbenoids in roots of non-infected and RKN-infected grapevines with Cabernet Sauvignon scion grafted to susceptible (O39-16) or resistant (Freedom) rootstocks. This was part of a larger effort to assess phenolic compound levels within grapevine rootstocks to determine roles of stilbenoid compounds in improving nematode resistance and overall plant health.

**Results:**

None of the assessed compounds were consistently greater in RKN infected plants versus healthy controls. Stilbenoids putatively identified as pallidol, ɑ-viniferin, miyabenol C, and hopeaphenol were overall much greater in Freedom than O39-16 rootstocks. By contrast, the stilbenoids ampelopsin A, ω-viniferin, and vitisin B were greater in O39-16 than Freedom. O39-16 and Freedom had similar levels of other stilbenoids especially monomers and dimers. Potentially the greater levels of specific stilbenoids present in Freedom than O39-16 provided RKN resistance. If validated, breeding programs could utilize the increased presence of these compounds as a marker for increased resistance to nematodes.

## Introduction

Root knot nematodes (RKN, *Meloidogyne* spp.) can be major pathogens almost everywhere grapevines are grown as populations can build up over time to severely affect root functioning, with effects on overall plant health and yields [1].

Most commercial grapevines are now *Vitis vinifera* cultivars grown as scions grafted onto rootstocks, as some of these rootstocks possess medium to high levels of resistance to RKN from breeding projects dating back to the 1950s [2–8]. The mechanisms for resistance to RKN remain unclear, with work to characterize how grapevines could ward off nematodes only beginning [4]. One potential mechanism is the production of a class of phenolic compounds called stilbenoids, which are mostly associated with being antibiotics against microbes [9]. A recent study of stilbenoids present in roots of grapevine was conducted on self-rooted ‘Cabernet Sauvignon’ and quantified high levels of five stilbenoid compounds: resveratrol, piceatannol, piceid, ε-vinifierin, and δ-viniferin [10].

However, more stilbenoids exist in grapevine rootstocks as multiple species can comprise specific ones. This is the first study to relate concentrations of stilbenoids to observed resistance that grapevine rootstocks may possess against nematodes. Thus, stilbenoid levels were assessed in a susceptible rootstock cultivar ‘O39-16′ (*V. vinifera* × *Vitis rotundifolia*) and a resistant rootstock cultivar ‘Freedom’ [*Vitis champinii* × (*Vitis solonis* x (*V. vinifera* × (*Vitis riparia* × *V. labrusca*)))]. Future and ongoing studies will examine additional rootstocks with different backgrounds. Findings could be used to aid the development of novel RKN resistance molecular markers for use in grapevine breeding efforts.

## Main text

### Materials and methods

#### Experimental design and sample collection

In both June of 2015 and 2016, a total of 16 for each of 2 year old ‘Cabernet Sauvignon’ grapevines either grafted to ‘O39-16′ (RKN-susceptible) or ‘Freedom’ (RKN-resistant) [7, 13] in 3 gallon pots were inoculated with a RKN, *Meloidogyne incognita* (Kofoid & White) Chitwood, by pipetting 10 mL of a nematode suspension, containing a total of 1000 nematode eggs, into the soil around the plants. The treatments were arranged as a completely randomized block design, with the plants kept in temperature-controlled greenhouse (about 22 °C to 32 °C), carefully watered weekly to avoid water flow-through, and received natural sunlight for the entire duration of the experiment. Four controls and four RKN infected plants were harvested at 6 and 12 weeks post-inoculation treatment. At each harvest, the plants were removed from the pots, with the roots briefly rinsed in water, and sampled by using pruning shears to collect six semi-randomly collected segments covering fine, lateral, and tap roots (roughly 10 g total were collected) for nematode extractions, and additional roots were collected similarly and flash-frozen in liquid nitrogen and kept at − 20 °C for compound extractions. The leftover soil was then hand-mixed with roughly 50 cm^3^ collected in a 50 mL centrifuge tube for soil nematode counts.

#### Root knot nematode counts

RKN counts were made in both the root tissues and collected soil. In brief, modified Baermann funnels were set up with filter paper, on which a weighed amount of roots were (roughly 5 g) submerged in water. The end of the funnel had a small amount of rubber tubing closed with a binder clip. Likewise, 50 cm^3^ of soil was measured out and placed on filter paper and submerged. After 48 h, the water was collected from the funnel assembly, and brought to 10 mL total. A 1 mL aliquot of this was then placed in a deep-well microscope slide with a 4 1 × 1 mm grid for counting RKN at the mobile juvenile (J2) stage. Final counts were adjusted to a per g root or per cm^3^ soil amounts.

#### Stilbenoid extraction and quantification

Chemical analyses proceeded based on modified methods of Wallis et al. [11] and Wallis and Chen [12]. All reagents and solvents were provided by Thermo-Fisher Scientific (Waltham, MA, USA). All frozen root samples, including some with galls, were pulverized with a mortar and pestle in liquid nitrogen and had three 0.10 g aliquots weighed out into three 1.5 mL centrifuge tubes and then extracted overnight at 4 °C in methanol. Remaining pellets were re-extracted in 0.5 mL of the same solvent, with this second extract combined with the first 1.0 mL total extract after combination.

High-performance liquid chromatography (HPLC) was used to examine stilbenoid compounds from these methanol extracts. A total of 50 µL of the methanol extract was injected into a Shimadzu (Columbia, MD, USA) LC-20AD pump based liquid chromatograph equipped with Supelco Ascentis RP-18 (Sigma-Aldrich, St. Louis, MO, USA) column and a Shimadzu PDA-20 photodiode array detector. Sigma-Aldrich provided piceatannol, resveratrol, and ε-viniferin, which were used to identify these compounds. Other compounds were identified via liquid chromatography-mass spectrometry using a Shimadzu LCMS2020 system [12] and comparing molecular weight information and relative retention times with those previously reported for grapevine stems and roots (Table 1). The obtained weights of phenolics present within samples were derived by running standard curves made using resveratrol [12].

**Table 1:** Compounds quantified in this study and criteria used for putative identifications

Stilbenoid type	Putative name	Retention time	Molecular weight	References
Monomer	Piceatannol	19.1	243	[14–16]
	Resveratrol	21.5	228	[14–16]
Dimer	Ampelopsin A	16.3	469	[14, 15]
	Ampelopsin D/ quadrangularin A	18.8	454	[17]
	ε-viniferin^a^	24.0	454	[10, 14–17]
	Pallidol	18.1	454	[17]
	ω-viniferin	26.6	454	[15, 16]
Trimer	α-viniferin	24.7	680	[17]
	Miyabenol C	22.3	680	[14, 15, 17]
Tetramer	Hopeaphenol^b^	25.9	906	[14, 15]
	Vitisin B	27.8	906	[15, 16]

^a^Compound potentially co-eluted with δ-viniferin

^b^Compound potentially co-eluted with isohopeaphenol

#### Statistical analyses

IBM (Armonk, NY, USA) SPSS statistics version 22, with α = 0.05, was used for all statistical analyses. Outliers consistently greater than two standard errors of a mean for each factor were excluded from analyses [11]. Unless stated, for all analyses N = 32.

Due to a lack of meeting normality assumptions, non-parametric Mann–Whitney U tests were used to confirm differences in RKN nematodes present within soil and roots among the grapevine rootstock cultivars (O39-16 or Freedom), for each sampling time (6 or 12 weeks), and for each year (2015 or 2016) for RKN-inoculated plants only, as all non-inoculated plants did not have RKN observed.

Analyses of variance were employed to compare differences in individual compounds between rootstock cultivars and differences due to inoculation status (with the interaction also in the model). Each year was treated as a separate experimental trial.

### Results and discussion

#### Nematode counts

**Fig. 1:**
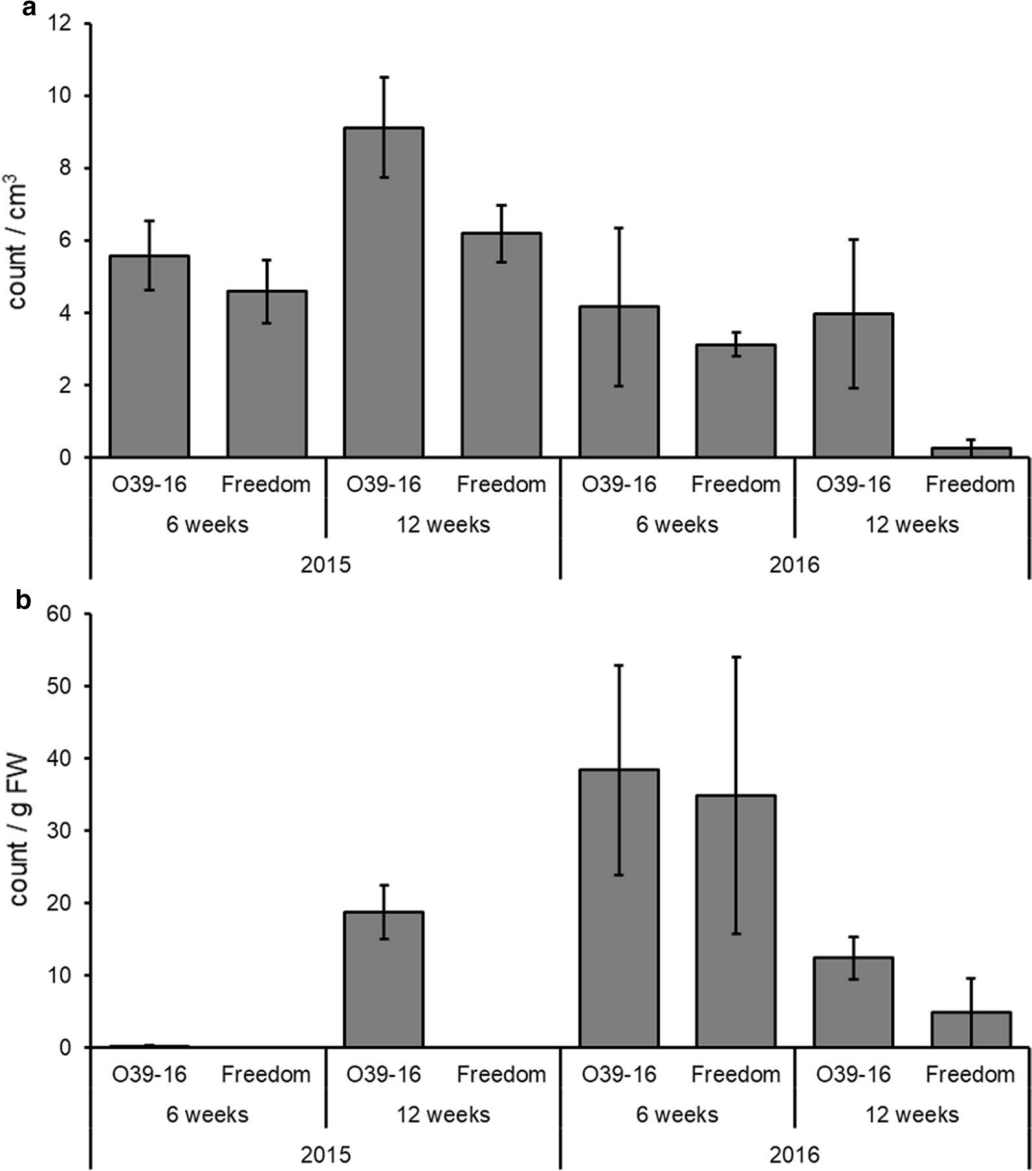
Mean nematode (J2 stage) counts (± SE) present in (**a**) soil or (**b**) roots of inoculated plants. *FW* fresh weight

Nematode levels, measured as J2 stage juveniles, in the soil were significantly less when the resistant ‘Freedom’ was used instead of susceptible ‘O39-16′ as the rootstock (Mann–Whitney U = 73.000; *P* = 0.038) (Fig. 1a). Fewer nematodes were counted in the soil in 2015 than 2016 (Mann–Whitney U = 50.500; *P* = 0.003). There were no differences in soil nematode counts between sampling done in week 6 compared with week 12 (Mann–Whitney U = 104.500; *P* = 0.375).

Nematode counts in the roots, measured as J2 juveniles collected via Baermann funnels, were greater in the susceptible ‘O39-16′ rootstocks than resistant ‘Freedom’ (Mann–Whitney U = 60.500; *P* = 0.009) (Fig. 1b). Nematode counts in root samples also were less in 2015 than 2016 (Mann–Whitney U = 62.500; *P* = 0.011). There was no a significant difference in root nematode counts when samples were collected at 6 versus 12 weeks (Mann–Whitney U = 113.500; *P* = 0.574).

Potential differences in nematode growth rates, due to variances in environmental conditions and pre-existing plant health, likely resulted in different counts between weeks and years. Future studies are warranted whereby nematode populations are more carefully measured over an increased time course, such as weekly or biweekly for several months, to appropriately assess fluxes in nematode counts over time.

However, these results likely confirm previous observations about resistance, as it was observed that ‘Freedom’ rootstocks possess some resistance to nematode infections whereas ‘O39-16′ rootstocks were susceptible [7, 13].

#### Root stilbenoid levels

A total of eleven stilbenoids were putatively identified in this study, and each compound was analyzed by week and year separately (Table 1). The stilbenoid compounds quantified in this study were similar as those found in other studies (Table 1), albeit the resveratrol glycoside piceid was not observed in quantifiable amounts, with only trace characteristic ions observed by LC–MS, despite being observed previously [10, 14]. However, piceid also was not present in sufficiently quantifiable amounts in *V. vinifera* by Lambert et al. [15] or in many of the wild *Vitis* spp. studied by Pawlus et al. [16]. The putatively identified miyabenol C, hopeaphenol, and ε-viniferin were the most prevalent stilbenoids observed in this study, which was like previous observations [10, 14, 15].

For most analyses, there were no significant differences due to infection status, with a few exceptions (Table 2). Nematode infections increased levels of piceatannol, ampelosin D/quandrangularin A, and ɑ-viniferin in week 12 of 2016. By contrast, ampelopsin A, ω-viniferin, and vitisin B were present in lower levels in nematode infected plants compared to controls during week 12 of 2016. Pallidol had greater levels in nematode infected plants compared to controls in week 6 of 2016. Despite inherit variability in this study in terms of nematode populations, the findings that certain stilbenoid compounds increased suggests some induction of these compounds occurred as a host response. As for the lack of other compounds from being affected by feeding, it could be hypothesized that RKN manipulations of host cells altered compound levels, albeit unevenly in the root samples of this study as both galled and ungalled tissues were analyzed. Further, RKN might possess mechanisms that reduced or altered host responses associated with herbivory.

**Table 2:** Mean (± SE) concentrations of individual stilbenoids (µg/g FW) in healthy or RKN-infected roots

				O39-16		Freedom			*F*-statistic	
Stilbenoid type	Putative name	Year	Week	Control	RKN	Control	RKN	Cultivar	Inoculation	Interaction
Monomer	Piceatannol	2015	6	17.6 ± 6.5	37.3 ± 17.0	12.5 ± 3.6	19.0 ± 4.0	1.521	1.912	0.490
			12	15.5 ± 2.7	75.6 ± 27.8	11.9 ± 2.9	17.6 ± 0.8	4.839*	5.502*	3.756
		2016	6	17.5 ± 4.0	35.0 ± 8.5	36.7 ± 6.0	30.2 ± 18.5	1.588	0.954	4.509
			12	26.4 ± 3.0	36.2 ± 11.8	31.8 ± 1.1	39.9 ± 3.7	0.337	1.709	0.001
	Resveratrol	2015	6	301 ± 99	1170 ± 660	105 ± 29	131 ± 43	3.383	1.770	1.567
			12	100 ± 27	738 ± 486	106 ± 13	111 ± 40	1.625	1.737	1.686
		2016	6	216 ± 27	323 ± 151	299 ± 79	192 ± 22	0.078	0.000	1.520
			12	281 ± 88	490 ± 226	204 ± 25	252 ± 14	1.659	1.112	0.434
Dimer	Ampelopsin A	2015	6	250 ± 51	265 ± 62	204 ± 44	281 ± 72	0.055	0.512	0.241
			12	255 ± 51	314 ± 67	235 ± 28	228 ± 28	1.211	0.293	0.469
		2016	6	451 ± 59	480 ± 45	408 ± 77	304 ± 36	3.804	0.449	1.393
			12	578 ± 47	307 ± 22	288 ± 26	324 ± 29	17.527**	13.049**	22.154***
	Ampelopsin D /quadrangularin A	2015	6	11.9 ± 3.8	24.6 ± 9.5	21.8 ± 4.6	27.8 ± 4.3	1.206	2.435	0.309
			12	10.2 ± 1.8	38.3 ± 13.0	23.3 ± 1.7	26.1 ± 1.4	0.005	5.356*	3.620
		2016	6	26.7 ± 7.5	32.0 ± 9.8	51.1 ± 3.7	51.7 ± 1.3	11.509**	0.209	0.133
			12	28.3 ± 5.4	30.0 ± 6.3	52.1 ± 6.9	58.6 ± 17.6	6.430*	0.156	0.054
	ε-viniferin	2015	6	350 ± 33	212 ± 88	218 ± 63	174 ± 56	1.833	2.092	0.567
			12	267 ± 91	223 ± 23	131 ± 9	181 ± 40	3.024	0.004	0.836
		2016	6	387 ± 55	372 ± 47	274 ± 49	234 ± 27	7.544*	0.360	0.076
			12	379 ± 9	214 ± 51	248 ± 18	331 ± 73	0.022	0.828	7.445*
	Pallidol	2015	6	69.5 ± 10.5	84.6 ± 20.7	182 ± 6	184 ± 40	20.386***	0.135	0.077
			12	61.3 ± 10.2	80.5 ± 18.4	141 ± 19	177 ± 32	17.415***	1.737	0.165
		2016	6	86.0 ± 18.6	127 ± 15	122 ± 13	149 ± 6	4.433	5.890*	0.255
			12	125 ± 18	104 ± 17	162 ± 20	197 ± 43	6.069*	0.070	1.112
	ω-viniferin	2015	6	247 ± 49	208 ± 26	83.4 ± 16.6	97.0 ± 21.7	20.006***	0.175	0.739
			12	358 ± 30	304 ± 43	103 ± 9	105 ± 13	68.739***	0.930	1.029
		2016	6	363 ± 51	459 ± 28	298 ± 75	204 ± 16	11.045**	0.001	3.911
			12	528 ± 46	312 ± 17	196 ± 14	241 ± 12	57.218***	10.148**	23.936***
Trimer	α-viniferin	2015	6	33.1 ± 2.4	27.9 ± 5.6	32.7 ± 5.8	36.8 ± 8.1	0.532	0.008	0.631
			12	41.2 ± 5.8	42.1 ± 5.1	62.9 ± 2.9	63.4 ± 4.2	21.407***	0.026	0.003
		2016	6	78.8 ± 5.5	63.2 ± 2.9	82.6 ± 6.9	99.5 ± 16.2	4.615	0.005	3.036
			12	53.3 ± 2.4	61.2 ± 5.5	74.5 ± 6.5	96.1 ± 5.5	28.269***	7.844*	1.661
	Miyabenol C	2015	6	6.39 ± 1.42	11.2 ± 7.4	148 ± 22	169 ± 24	53.106***	0.411	0.165
			12	6.62 ± 0.90	19.2 ± 4.9	187 ± 9	189 ± 29	101.094***	0.171	0.096
		2016	6	69.8 ± 52.5	11.1 ± 2.8	190 ± 60	219 ± 18	16.031**	0.128	1.155
			12	8.67 ± 1.30	11.3 ± 2.1	216 ± 15	235 ± 1	805.294***	1.923	1.094
Tetramer	Hopeaphenol	2015	6	246 ± 45	176 ± 19	875 ± 224	1032 ± 300	15.514**	0.053	0.362
			12	352 ± 36	266 ± 41	884 ± 112	879 ± 167	30.134***	0.189	0.149
		2016	6	596 ± 207	439 ± 19	1100 ± 230	1260 ± 120	16.355**	0.000	0.913
			12	423 ± 39	282 ± 20	1140 ± 70	1290 ± 30	388.179***	0.159	16.672**
	Vitisin B	2015	6	81.6 ± 17.3	69.7 ± 13.1	8.79 ± 1.36	9.70 ± 2.08	50.634***	0.345	0.468
			12	131 ± 11	104 ± 18	9.81 ± 1.12	12.2 ± 1.9	104.258***	1.334	1.910
		2016	6	113 ± 32	162 ± 10	57.0 ± 38.1	17.3 ± 2.7	15.611**	0.033	3.044
			12	185 ± 19	105 ± 5	17.2 ± 1.4	20.1 ± 1.3	167.010***	15.505**	17.961**

*P < 0.05; **P < 0.01; ***P < 0.001

The susceptible ‘O39-16′ rootstocks consistently possessed greater levels of ω-viniferin and vitisin B than the resistant ‘Freedom’ rootstock (Table 2). By contrast, ‘Freedom’ rootstocks consistently possessed greater levels of miyabenol C and hopeaphenol (Table 2). Previously, Lambert et al. [15] observed vast differences in stilbenoid concentration among many *V. vinifera* cultivars, including the presence or virtual absence of certain compounds such as miyabenol C and vitisin B. Furthermore, Pawlus et al. [16] observed differences and presence or absence of certain stilbenoids among wild *Vitis* spp. as well. Unlike this study, Pawlus et al. [16] did not examine currently available commercial rootstock cultivars. Furthermore, although Lambert et al. [15] and Pawlus et al. [16] observed chemistry of stem tissues, this study determined similar differences when comparing stilbenoid levels in the roots of different species-derived rootstocks, namely large differences in certain specific compounds.

### Conclusions

Based on these observations, a hypothesis can be formed that miyabenol C and hopeaphenol levels potential impart resistance to RKN, as these compounds were present in levels four- to ten-fold greater in resistant ‘Freedom’ rootstocks than ‘O39-16′. By contrast, it seems that many stilbenoid monomers and dimers are not involved in RKN resistance, as otherwise greater levels of dimers (such as ε-viniferin and ω-viniferin) would make ‘O39-16′ more resistant. It could be hypothesized that ‘Freedom’ possesses enzymes that were more effective at producing stilbenoid trimers and tetramers than ‘O39-16′. Targeting genes responsible for producing stilbenoid polymer synthases could reveal genetic differences between the two cultivars and may be mapped as molecular markers of RKN resistance. These new markers could prove valuable in breeding efforts to impart RKN resistance in newly developed rootstocks.

## Limitations

This data set is limited by including only two cultivars, just one resistant and one susceptible, so firm conclusions about the roles of stilbenoids cannot be made at this time. Additional studies across a broader spectrum of both RKN susceptible and resistant rootstocks, and possible crosses between these rootstocks, would be necessary to support conclusions. Likewise, this study likely did not use a large enough inoculum to conduct this experiment- future studies should be inoculated with contaminated soil or roots to provide a greater RKN population and a variety of different life stages. Furthermore, sampling should be at an increased interval in future studies to capture fluctuations in RKN populations over time, perhaps incorporating weekly or biweekly sampling. Assessment of nematodes also should include gall counts/disease assessments as well to provide a more accurate picture of effects on host health. Other compounds and defense proteins also are likely involved in host defense against RKN. There also is the possibility that nutritional differences or unmeasured effects on overall plant health that differ between rootstock cultivars also could result in observed differences in RKN susceptibility. Lastly, bioassays that directly or indirectly observe the effects of stilbenoids on nematode reproduction, feeding, or survival would be necessary to support the hypotheses that certain compounds impart resistance. Unfortunately, the major of stilbenoid compounds are not commercially available, and time-consuming isolations or syntheses are needed for these studies to proceed.

## Data Availability

The data described in this Data note can be freely and openly accessed on the USDA Ag Data Common (https://data.nal.usda.gov/dataset/grapevine-rootstock-stilbenoid-data-and-rkn-induction).

## Abbreviation

RKN: Root knot nematode
